# COVID-19 in Brazilian Pediatric Patients: A Retrospective Cross-Sectional Study with a Predictive Model for Hospitalization

**DOI:** 10.3390/life14091083

**Published:** 2024-08-29

**Authors:** Ana Paula Pacheco, Henrique Laureano, Laire Schidlowski, Natalia Ciorcero, Thalita Zanatto, Ariela Borgmann, Gabrielle Fragoso, Ana Luisa Giamberardino, Renata Dourado, Karine dos Anjos, Paulo João, Marina Assahide, Maria Cristina Silveira, Victor Costa-Junior, Heloisa Giamberardino, Carolina Prando

**Affiliations:** 1 Programa de Pós-Graduação em Biotecnologia Aplicada à Saúde da Criança e do Adolescente, Faculdades Pequeno Príncipe, Curitiba 80230-020, PR, Brazil; 2 Instituto de Pesquisa Pelé Pequeno Príncipe, Curitiba 80250-060, PR, Brazil; 3 Serviço de Epidemiologia e Controle de Infecção Hospitalar, Hospital Pequeno Príncipe, Curitiba 80250-060, PR, Brazil; 4 Faculdades Pequeno Príncipe, Curitiba 80230-020, PR, Brazil; 5 Medical School, Faculdades Pequeno Príncipe, Curitiba 80230-020, PR, Brazil; 6 Residency in Pediatrics, Hospital Pequeno Príncipe, Curitiba 80250-060, PR, Brazil; 7 Laboratório Genômico, Hospital Pequeno Príncipe, Curitiba 80250-060, PR, Brazil; 8 Serviços Diagnósticos, Hospital Pequeno Príncipe, Curitiba 80250-060, PR, Brazil; 9 Unidade de Terapia Intensiva, Hospital Pequeno Príncipe, Curitiba 80250-060, PR, Brazil; 10 Serviço de Infectologia Pediátrica, Hospital Pequeno Príncipe, Curitiba 80250-060, PR, Brazil; 11 Unidade de Terapia Intensiva e Pronto Atendimento, Hospital Pequeno Príncipe, Curitiba 80250-060, PR, Brazil; 12 Centro de Vacinas, Hospital Pequeno Príncipe, Curitiba 80250-060, PR, Brazil; 13 Serviço de Alergia e Imunologia, Hospital Pequeno Príncipe, Curitiba 80250-060, PR, Brazil

**Keywords:** SARS-CoV-2, pediatrics, hospitalization

## Abstract

Background: This study was conducted to ascertain the most frequent symptoms of COVID-19 infection at first consultation in a pediatric cohort and to devise a predictive model for hospitalization. Methods: This is a retrospective cross-sectional study of 1028 Brazilian patients aged <18 years with SARS-CoV-2 infection in a single reference hospital in the first year of the pandemic. Clinical, demographic, laboratory, and disease spectrum data were analyzed via multivariate logistic regression modeling to develop a predictive model of factors linked to hospitalization. Results: The majority of our cohort were schoolchildren and adolescents, with a homogeneous distribution concerning sex. At first consultation, most patients presented with fever (64.1%) and respiratory symptoms (63.3%). We had 204 admitted patients, including 11 with Pediatric Multisystem Inflammatory Syndrome. Increased D-dimer levels were associated with comorbidities (*p* = 0.018). A high viral load was observed in patients within the first two days of symptoms (*p* < 0.0001). Our predictive model included respiratory distress, number and type of specific comorbidities, tachycardia, seizures, and vomiting as factors for hospitalization. Conclusions: Most patients presented with mild conditions with outpatient treatment. However, understanding predictors for hospitalization can contribute to medical decisions at the first patient visit.

## 1. Introduction

SARS-CoV-2 emerged in China at the end of 2019, causing COVID-19 disease, with a varied spectrum, from asymptomatic to Severe Acute Respiratory Syndrome (SARS). In mid-March 2020, owing to its global spread, it was declared a pandemic by the World Health Organization. COVID-19 can present a wide spectrum of clinical symptoms, ranging from asymptomatic to severe conditions corresponding to less than 1% of cases [1,2,3].

During the first year of this pandemic, approximately 84,586,904 people worldwide were affected, with 1,835,788 deaths. Brazil was the third highest country in number of confirmed cases of SARS-CoV-2 in this period, with 7,716,405 cases and 149,435 deaths [4]. The southeastern region had the highest absolute number of cases (2,703,086), with the highest incidence in the midwestern region (5383.6 per 100,000 inhabitants). In the southern region, there were 1,369,059 cases, with an incidence in this period of 4567.2 per 100,000 inhabitants [5]. Children accounted for about 2% of COVID-19 cases in China, 1.2% in Italy, and 5% in the United States [1,2,3]. In Brazil, in 2020, 2.46% of all COVID-19 cases were in children, with schoolchildren and adolescents (6–19 years old) corresponding to 48.3% of cases in children under 19 years old [5].

COVID-19 in children is generally less severe and has a lower lethality than in adults [6,7]. However, with the progression of the global pandemic, severe and life-threatening manifestations of the disease have emerged in pediatric patients, including Pediatric Multisystem Inflammatory Syndrome (P-SIM) [7,8]. The low incidence of COVID-19 in children may be due to a lower susceptibility to or milder or asymptomatic forms of the disease [1,2].

The objective of this study was to consolidate clinical and laboratory information for 1028 pediatric patients infected with SARS-CoV-2 at the moment they presented for their first medical consultation in a single reference center located in the southern region of Brazil, as well as to define, based on this presentation, and to develop a predictive model for, factors related to the risk of hospitalization to bring more subsidies to support medical decisions.

## 2. Materials and Methods

This cross-sectional, retrospective observational study was conducted in a pediatric quaternary care hospital, a reference center for the care of children and adolescents with COVID-19 in the southern region of Brazil. This study was approved by the Ethics and Research Committee (Number: 35870420.1.0000.0097).

The study included data from 1028 patients not vaccinated for COVID-19, aged <18 years, with a laboratory diagnosis of COVID-19, treated from 1 January 2020 to 30 June 2021, based on notifications from the Hospital Epidemiological Surveillance Center (NVEH).

Electronic medical records of the participants were reviewed and information was collected, including demographic variables, clinical signs of first care, comorbidities, laboratory parameters, radiological findings, previous contact with suspected or confirmed cases of COVID-19, and hospitalization. Disease was classified as asymptomatic, mild (no need for hospitalization), moderate (hospitalization in the ward), or severe (hospitalization in the intensive care unit [ICU]). For information presented in this study where it was not possible to provide data for all cases, the final number is indicated at each stage of the results.

To summarize the data, we calculated the means, standard deviations, frequencies, percentages, and proportions. The variables were selected according to the presentation of COVID-19 in pediatrics at the time, considering signs/symptoms and laboratory and imaging results. For the variables that were normally distributed, if the assumption of normal distribution was not rejected, a parametric hypothesis test was used. If the assumption of normal distribution was rejected, a corresponding nonparametric test was used. Several graphs were created during the exploratory analysis to understand the data. By crossing categorical variables and examining their numerical outputs in other variables, we gained a better understanding of how the data related and could identify hidden behaviors and relationships. Differences between clinical profiles were statistically tested using Welch’s *t*-test and Pearson’s chi-square test. Based on a representative number of measured patient characteristics, predictive statistical models [9] were designed to probabilistically classify a patient in terms of the following desired outcome: the need for hospitalization (ward or ICU).

A multivariate logistic regression model was used with a stepwise variable selection procedure in both directions (forward and backward) based on the Akaike Information Criterion (AIC) [10]. For development of the model, the data were split into training and testing sets, with 80% for training and 20% for testing, using a 10-fold cross-validation procedure on the training set [10]; more details are available in the Supplementary Materials. All analyses were performed using R software, version 4.3.2. The main R libraries used were {dplyr}, {tidyr}, {forcats}, {lubridate}, {stringr}, {purrr}, {MASS}, {marginaleffects}, {ggplot2}, and {patckwork} [9,10,11,12,13,14,15,16,17,18,19,20].

## 3. Results

**Figure 1:**
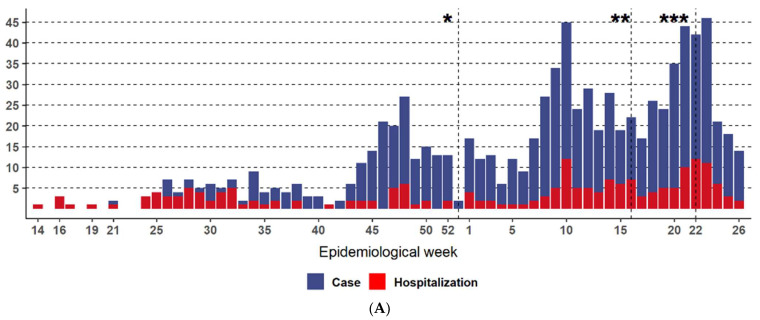

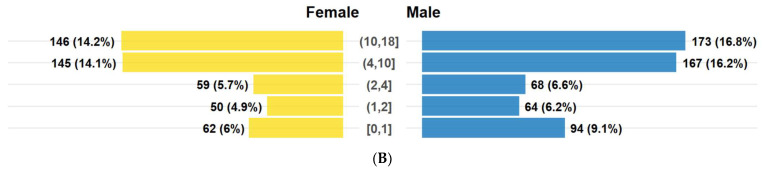
(**A**) Distribution of 1028 COVID-19 cases treated at a quaternary pediatric hospital by epidemiological week, showing total numbers of cases in blue and admissions in red. (**B**) Proportion of COVID-19 cases by sex and age. (*) Gamma variant in Paraná and Curitiba, (**) Delta variant in Paraná, (***) Delta variant in Curitiba.

This study analyzed 1028 pediatric patients with a laboratory diagnosis of COVID-19 who were treated at a single pediatric hospital. The cases occurred between April 2020 and June 2021 (epidemiological weeks 15 to 53/2020 and 01 to 26/2021), with a higher number of new cases and hospitalization in June 2021 (epidemiological weeks 23 to 26/2021) (Figure 1A). A total of 983 (95.62%) patients were residents of either Curitiba (n = 659) or the metropolitan region (n = 324), while 33 (3.2%) were from other cities in Paraná, and 12 (1.2%) were from other states of Brazil. The age of the patients ranged from 0 to 17.8 years (mean 6.8 years, median 6.29 years), with a predominance of schoolchildren and adolescents. The sample was homogeneous in terms of sex (462 females and 566 males) (Figure 1B).

**Figure 2:**
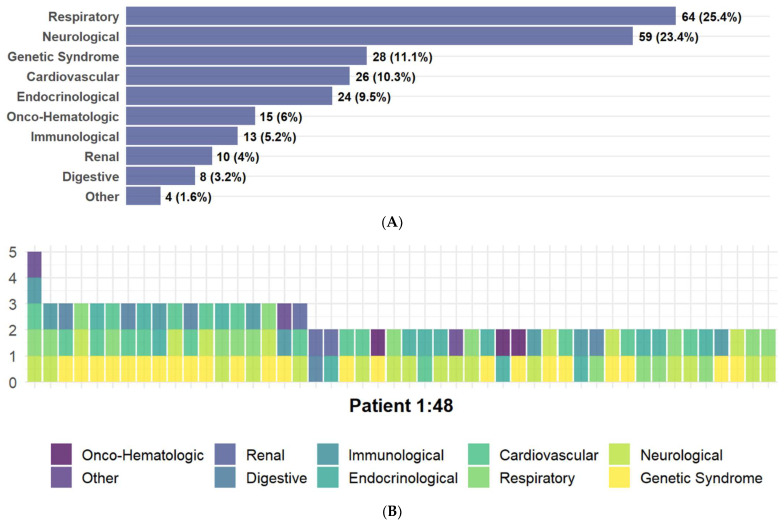
(**A**) Pre-existing conditions in 183 of 1028 patients with COVID-19. (**B**) Combination of pre-existing conditions in 48 patients.

One hundred and eighty-three (17.8%) patients had comorbidities. The most frequent comorbidities were related to pathologies of the respiratory and neurological systems (Figure 2A). A combination of more than one comorbidity was present in 48 patients (4.7%) (Figure 2B). The mean age of patients with comorbidities was 8.05 years, and this was significantly different from that of the group without comorbidities (mean age 6.64 years) (*p* = 0.0012).

In total, 204 (19.8%) patients were hospitalized, and among them, 108 (52.9%) had comorbidities. ICU patients (n = 60) accounted for 5.8% of the total cohort, 29.4% of whom were hospitalized. When we assessed each year of age individually, we found no significant difference in the need for hospitalization. The presence of comorbidities was higher among hospitalized patients (*p* < 0.0001).

Regarding the presence of symptoms, 995 (96.7%) patients were symptomatic, including 16 patients who developed symptoms after the first assessment (time to symptom onset, 1 to 10 days). For 782 participants of this study, we were able to obtain information about previous contact with a suspected or confirmed case of COVID-19, and 737 (94.2%) participants, including 29 asymptomatic participants, reported such contacts. It was possible to evaluate the time elapsed between symptoms and diagnosis for 861 of the patients, with an established average of 2.66 days (range −10 to 34 days). In 829 (96.2%) patients, the diagnosis was made within 1 week of the onset of the first symptoms.

Regarding the presentation of the disease, we classified symptoms into respiratory, digestive, and general. Most children and adolescents with COVID-19 had respiratory disease (63.3%; 681/1028), of whom 15.7% (107/681) required hospitalization, followed by 19.4% (199/1028) with general symptoms, 10.1% (104/1028) with digestive symptoms, 3.21% (33/1028) with asymptomatic presentation, and 1.1% (11/1028) that were diagnosed with P-SIM.

In general, the most common clinical manifestations in the first visit were fever (64.1%) and cough (40.1%), followed by runny nose (29.6%), headache (19.5%), odynophagia (16.8%), diarrhea (12.5%), difficulty breathing (12%), vomiting (9.6%), lack of appetite (9.5%), myalgia (7.5%), sneezing (6.2%), nasal obstruction or congestion (5.6%), abdominal pain (5.5%), and nausea (4.7%). Other signs and symptoms appeared in less than 3% of cases (Table 1). A total of 13.5% of the patients who had a fever and 8.4% of those who had a cough required hospitalization. Of all patients who had tachypnea, 93.7% of those who had seizures and 80% of those who had tachycardia were hospitalized. Among the 11 patients diagnosed with SIMP, fever (90.9%), abdominal pain, and vomiting (54.6%) were the most frequent symptoms.

During the initial visit, blood counts were conducted for 301 patients as part of the laboratory tests. Of these, 197 required hospitalization. Considering the values corrected for age, 175 (58.1%) participants had normal lymphocyte count, 118 (39.2%) had lymphopenia, and only 8 (2.7%) had lymphocytosis. While 63 (20.9%) participants had neutropenia, 59 (19.6%) had neutrophilia, and 179 (59.5%) had a normal neutrophil count.

C-reactive protein (CRP) levels were measured in 299 patients (29%). In 202 patients (67.6%), the CRP level increased, with a mean value of 39.4 mg/dL. A total of 173 participants underwent D-dimer level testing, of whom 164 (94.8%) were hospitalized. Ninety-four (54.3%) patients had altered D-dimer levels, with a mean of 1044 ng/mL and a median of 536 ng/mL. Patients with associated comorbidities had higher values for both CRP (*p* = 0.049) and D-dimer (*p* = 0.018) levels.

By employing a qRT-PCR test for SARS-CoV-2, it was possible to assess the cycle threshold in 810 participants. Cycle threshold values below 22 were designated as a high viral load, values between 22 and 28 were considered a medium viral load, and values above 28 were considered a low viral load. Most patients (54.5%) had cycle threshold findings corresponding to a high viral load. It was observed that the higher the viral load, the shorter the time between the onset of symptoms and the search for medical care, with an average time of 2.06 days for a high viral load, 3.07 days for a medium viral load, and 3.90 days for a low viral load (*p* < 0.0001).

The imaging methods used for evaluations were radiography and chest computed tomography (CT). Of the 225 participants who underwent radiography, 141 (62.7%) had a normal report and 84 (37.3%) had an abnormal report (interstitial infiltrate, n = 14; condensation, n = 39; others, n = 31). In total, 110 patients underwent chest CT, of whom 91 (82.7%) showed alterations in the chest area, with ground-glass opacification being the most prevalent, being present in 69 (75.9%) cases.

**Figure 3:**
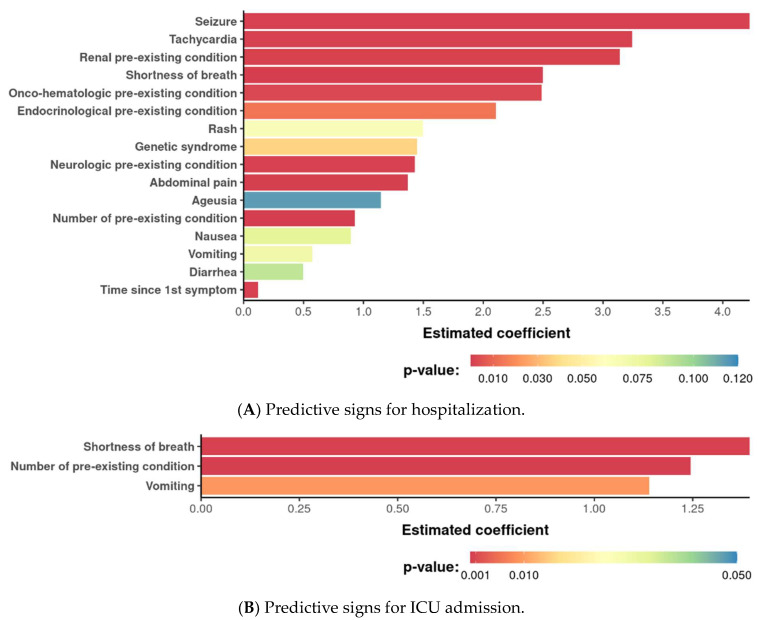
Predictors of hospitalization in a pediatric COVID-19 cohort. The final logistic regression model that predicts the risk of hospitalization is shown (**A**), including more specifically ICU admission (**B**). Each bar represents the coefficient estimated by the adjusted force.

A predictive model based on multivariate logistic regression considered all variables included in the study according to AIC scaling, identifying those that correlated with hospitalization outcomes. In this context, seizure, tachycardia, pre-existing renal condition, respiratory distress, and pre-existing onco-hematological condition were the five strongest predictors of hospitalization (Figure 3A). Respiratory distress was the strongest predictor, along with the number of pre-existing conditions and vomiting, for ICU admission (Figure 3B). For each model, the impact of the variable was estimated so that it was possible to calculate the probability of the outcome for each patient. The accuracies of the hospitalization and ICU models were 88.8% and 76%, respectively.

## 4. Discussion

In this retrospective cross-sectional study of 1028 Brazilian children with SARS-CoV-2 infection presenting to a single reference hospital for treating this infection, we reviewed clinical, demographic, and laboratory data and characterized the disease spectrum. We also identified the predictors of hospitalization in the pediatric population during the first year of the COVID-19 pandemic.

Although our data show a higher number of hospitalized children than in previous studies [21,22], most presented with a mild condition without the need for hospitalization in the first consultation, corroborating earlier findings that children have less severe COVID-19 than adults [6,7].

The clinical presentation of the disease in our study was similar to that reported in other pediatric studies, with fever and cough as the main initial symptoms [22,23,24]. For comparison with Brazilian data, another study that also evaluated children with and without the need for hospitalization found that fever, cough, and runny nose were the most frequent symptoms, similar to what was found in our study [22]. Hospitalized patients had a similar clinical presentation, with fever, cough, and difficulty breathing as the main symptoms. Tachycardia and seizures, which were significant in our study participants, have not been reported for other groups of pediatric patients with COVID-19. This difference may be related to the methodologies used in these studies [7,25], in which the data were obtained from the Influenza Epidemiological Surveillance Information System (SIVEP), a database that does not include direct acquisition of tachycardia or seizures. Concerning seizures, our hospital is a reference center for the care of rare and complex diseases in the country. Therefore, we assisted more children in the study cohort who had comorbidities, including neurological ones.

Excluding general symptoms, respiratory (63.3%) and digestive (10.1%) complaints were the most frequent, as has been verified in other studies [22,23]. Although children usually have mild forms of the disease, 19.8% (204/1028) of our study cohort were hospitalized, including 11 patients (1.1% of the total, 5.4% of those hospitalized) diagnosed with Pediatric Multisystem Inflammatory Syndrome (P-SIM), and this reflects the spectrum seriousness of the disease. Of the patients with P-SIM, more than 90% had fever and more than half presented with gastrointestinal tract symptoms (abdominal pain and vomiting), as has also been observed by [26] in a systematic review that included 440 patients.

Similar to the case with previous reports, we found that more than 90% (737/782) of our study participants experienced contact with a suspected or confirmed case of COVID-19, showing that the children were not the index cases and that they were usually infected at home or in other close-contact situations [22,27,28].

The average time from the onset of symptoms to diagnosis was 2.7 days (range −10 to 34 days), similar to the average time found in a study that included more than 11,000 Brazilian children [7]. In 96.2% (829/861) of our study cohort, the diagnosis was made less than 1 week after the onset of the first symptoms, and this may reflect the ease of access to healthcare and diagnosis in our region. It is worth mentioning that the study was conducted in a capital city in the south of the country with its own laboratory for the diagnosis of COVID-19, and it is not possible to make generalizations from these data.

In adults, lymphopenia and increased D-dimer levels are known to be associated with severity of COVID-19 symptoms [29]. Our data, as well as those of others [23,30], revealed no association between lymphopenia and COVID-19 symptom severity. Regarding D-dimer levels, half of the patients in our study who were tested showed changes in this parameter. Increased levels of this marker have also been reported in severe pediatric COVID-19 cases [31]. It was still possible to establish a relationship between the presence of associated comorbidities and higher D-dimer levels in our study participants, indicating an association between pre-existing conditions and signs of COVID-19 severity.

Consistent with previous reports, we observed that pediatric COVID-19 patients may have a high viral load, especially in the early days of infection [32]. In our study, the average time between the onset of symptoms and diagnosis was 2 days for patients with a high viral load and twice as long for patients with a low viral load. Assessing the viral load of more than 800 participants was differential in our study, possibly because the study site is a hospital complex that has its own laboratory that responded efficiently to the demand for these assessments, including an RT-PCR test for SARS-CoV-2 at the beginning of the pandemic.

Chest radiography, despite being more accessible, was not a good imaging modality for COVID-19 in our group, as almost two-thirds (62.7%) of these examinations showed normal results, while most (82.7%) of the chest CT scan results showed changes, with ground-glass opacification being the most prevalent finding, similar to that reported by others [23,33]. By reviewing data from hospitalized Brazilian children in the literature, we noted a higher proportion of changes observed via chest tomography in our study (91.7%) against 66.7% in a group from the city of São Paulo [22], while ground-glass opacification was the main change reported in both studies.

Based on the data obtained during the first consultation for 1028 Brazilian pediatric patients with COVID-19, the predictive model we developed allowed us to predict the need for hospitalization more accurately than for ICU admission. Respiratory distress was a strong predictor in our study and also in another study that developed a predictive model for hospitalization based on data from 1000 pediatric patients with COVID-19 [21]. The comorbidities present in 17.8% of our patients appeared to be predictive factors for hospitalization in the model we developed. Although respiratory and neurological comorbidities were the most frequent, renal and onco-hematological comorbidities were separately identified as predictors of hospitalization. What makes our model different is that in addition to identifying comorbidities as a predictive factor, we were able to define the type of comorbidity that could impact hospitalization outcomes, and we postulated that the number of comorbidities determined the need for ICU admission. Other exclusive predictors identified in our study included seizures and tachycardia. Unlike Howard et al. [22], we did not collect data on race and ethnicity, which appeared to be predictors of hospitalization in the cohort of the earlier study.

Despite the significant number of children included, this is the experience of a single center with highly complex characteristics, which may limit the generalizability of the results. The single center allowed for uniformity in data collection and viral load assessments, which were performed in the hospital laboratory. However, as this was a retrospective study, we also found limitations in data collection, such as the exclusion of the ethnic information of participants and its association with socioeconomic factors. Also, we did not collect data related to O2 saturation. The patients studied were treated in an emergency room or hospitalized at a reference hospital in the city, and our data may reflect the clinical manifestations of the most affected and/or those with greater access to health services and the possibility of diagnosis. Despite these limitations, to our knowledge, this study included the largest number of children and adolescents from a single hospital center in the first consultation for COVID-19. Our data may be useful for managing resources and devising future policies for the care and follow-up of children and adolescents with COVID-19, including the assessment of predictive factors for hospitalization.

## Figures and Tables

**Table 1:** List of symptoms presented in 1028 single-center cases of pediatric SARS-CoV-2 infections.

Symptom	N Total	%	Outpatient	%	Inpatient	%
Respiratory						
Cough	412	40.08	326	31.71	86	8.37
Runny nose	304	29.57	253	24.61	51	4.96
Odynophagia	173	16.83	161	15.66	12	1.17
Difficulty breathing	123	11.96	42	4.09	81	7.88
Sneezing	64	6.23	55	5.35	9	0.88
Nasal obstruction or congestion	58	5.64	49	4.77	9	0.88
Hoarseness	14	1.36	13	1.26	1	0.10
Tachypnea	5	0.49	0	0.00	5	0.49
Gastrointestinal						
Diarrhea	129	12.55	92	8.95	37	3.60
Vomiting	99	9.63	62	6.03	37	3.60
Abdominal pain	57	5.54	35	3.40	22	2.14
Nausea	48	4.67	34	3.31	14	1.36
General						
Fever	659	64.11	520	50.58	139	13.52
Headache	200	19.46	179	17.41	21	2.04
Lack of appetite	98	9.53	76	7.39	22	2.14
Myalgia	77	7.49	64	6.23	13	1.26
Ageusia	21	2.04	16	1.56	5	0.49
Anosmia	20	1.95	16	1.56	4	0.39
Seizures	16	1.56	1	0.10	15	1.46
Rash/exanthem	12	1.17	4	0.39	8	0.78
Tachycardia	10	0.97	2	0.19	8	0.78

## Data Availability

The data presented in this study are available on request from the corresponding author due to privacy.

## Supplementary Materials

The following supporting information can be downloaded at: https://www.mdpi.com/article/10.3390/life14091083/s1, Figure S1: Calculation of the accuracy for the outcomes: hospitalization and need for ICU.
