# Supplementary material for: COVID-19 in Brazilian Pediatric Patients: A Retrospective Cross-Sectional Study with a Predictive Model for Hospitalization
Source: Life (Basel). 2024 Aug 29;14(9):1083. doi: 10.3390/life14091083 (PMC11433062; doi:10.3390/life14091083)

The final model described in our study, we estimated coefficients for each covariate/feature level, and based on that, we generated a forecast with the following performance metrics: accuracies, sensitivities (true positive rate), and specificities (true negative rate) [11].

Figure S1. Calculation of the accuracy for the outcomes: hospitalization and need for ICU

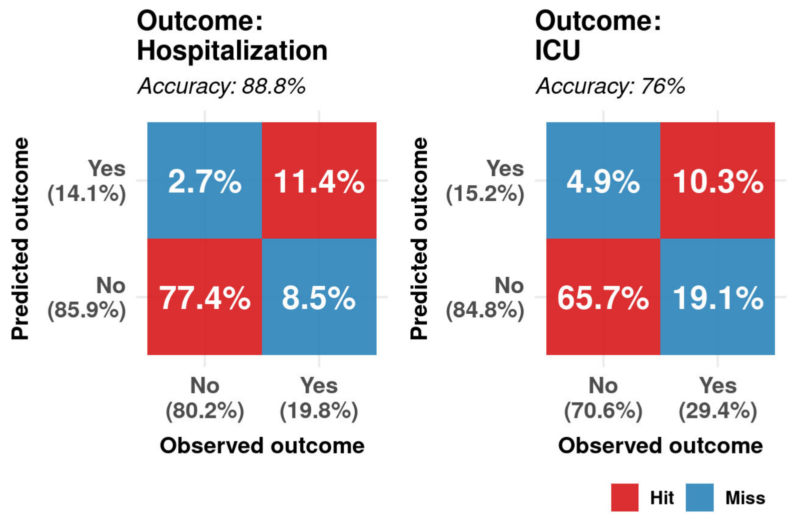

Supplement: Supplementary file 1 [file life-14-01083-s001.zip › life-3101839-supplementary.pdf]
